# Optimized patient transfer using an innovative multidisciplinary assessment in the Kanton Aargau (OPTIMA I): an observational survey in lower respiratory tract infections

**DOI:** 10.1186/cc9876

**Published:** 2011-03-11

**Authors:** F Dusemund, W Albrich, K Rüegger, R Bossart, K Regez, U Schild, A Conca, P Schuetz, T Sigrist, A Huber, B Reutlinger, B Müller

**Affiliations:** 1 Medical University Department of the University of Basel, Kantonsspital Aarau, Switzerland; 2 Department of Nursing, Kantonsspital Aarau, Switzerland; 3 Harvard School of Public Health, Boston, MA, USA; 4 Department of Pneumology, Kantonsspital Zug, Switzerland; 5 Department of Laboratory Medicine, Kantonsspital Aarau, Switzerland

## Introduction

Current medical scores have limited efficiency and safety to assign the most appropriate treatment site to patients with lower respiratory tract infections (LRTIs) [1-4]. We describe our current triage practice and assessed the potential of a combination of CURB65 with proadrenomedullin (ProADM) levels for triage decisions.

## Methods

Consecutive patients with LRTIs were prospectively followed and retrospectively classified according to CURB65 and ProADM levels (CURB65-A). Low medical risk patients were further subgrouped according to biopsychosocial and functional risks. We compared proportions of patients virtually allocated to triage sites with actual triage decisions and assessed the added impact of ProADM in a subgroup.

## Results

Ninety-six percent of 253 patients were hospitalized. Among the 138 patients with available CURB65-A, 17.4% had low medical risk indicating possible treatment in an outpatient or nonacute medical setting; 34.1% had intermediate medical risk (short hospitalization); and 48.6% had high medical risk (hospitalization). Fewer patients were in a low CURB65-A class (I) than a low CURB65 class (0, 1) (17.4% vs. 44.6%, *P *< 0.001). Mean length of hospitalization was 9.4 days including 3.5 days after reaching medical stability. In 56.9% of patients, hospitalization was prolonged after medical stability mainly for medical reasons.

## Conclusions

Current rates of hospitalization are high in patients with LRTI and the length of stay frequently extended beyond time of medical stabilization. The lower proportion of patients reclassified as low risk by adding ProADM to the CURB65 score might improve confidence in the triage algorithm.
